# Cumulative Dietary Risk of Pesticide Mixtures in Plant-Based Foods: Beyond Single-Compound Regulatory Compliance

**DOI:** 10.3390/foods15162868

**Published:** 2026-08-17

**Authors:** Gabriel Henrique Savietto, Ana Beatriz Cintra Da Silva, Pedro Henrique Da Silveira Neves, Lucas Silva Brito, Bruno Alves Rocha, Jonas Augusto Rizzato Paschoal, Joaquim Rovira, Jose L. Domingo, Fernando Barbosa, Marília Cristina Oliveira Souza

**Affiliations:** 1 Department of Biomolecular Sciences, School of Pharmaceutical Sciences of Ribeirão Preto, University of São Paulo, Av. do Café s/nº, Ribeirão Preto 14040-903, SP, Brazilmcosouza@usp.br (M.C.O.S.); 2 Institute of Chemistry, Federal University of Alfenas, Alfenas 37130-001, MG, Brazil; 3 Laboratory of Toxicology and Environmental Health, School of Medicine, Universitat Rovira I Virgili, Sant Llorenç 21, 43201 Reus, CT, Spain; 4 Institut de Recerca Biomèdica Catalunya Sud (IRB CatSud), 43204 Reus, CT, Spain; 5 Department of Clinical Analyses, Toxicology, and Food Sciences, School of Pharmaceutical Sciences of Ribeirão Preto, University of Sao Paulo, Av. do Café s/nº, Ribeirão Preto 14040-903, SP, Brazil

**Keywords:** dietary exposure, cumulative risk assessment, pesticide mixtures, maximum residue limits, fipronil, food safety

## Abstract

Dietary exposure to pesticide mixtures is recognized as an important food safety challenge worldwide, particularly in countries with intensive agricultural production. Regulatory monitoring programs focus primarily on compliance with maximum residue limits (MRLs), whereas cumulative exposure to pesticide mixtures remains insufficiently addressed. This study investigated the occurrence of eleven currently used pesticides in 20 commonly consumed plant-based foods (60 samples) marketed in Brazil and assessed regulatory compliance and human health risks associated with dietary exposure. Residues were determined by gas chromatography–mass spectrometry, and concentrations were compared with Brazilian MRLs and crop authorization status. Risk characterization included the hazard quotient (HQ), the hazard index (HI), and the carcinogenic risk (CRisk), estimated using cumulative assessment groups based on shared toxicological targets. Multiple residues were detected across all commodities, with chlorpyrifos, pirimiphos, imidacloprid, malathion, and fipronil most frequently associated with non-compliant samples. Several residues corresponded to pesticides not authorized for the respective crops, suggesting misuse, environmental contamination, or compliance failures; imidacloprid exceeded its MRL in zucchini by more than 70-fold. The carcinogenic risk from atrazine remained below accepted thresholds. In contrast, cumulative non-carcinogenic risk identified cassava, strawberry, and spinach as the commodities of greatest concern, with an HI of 3.16 for cassava. Fipronil residues drove these results, accounting for 83–99% of the cumulative hazard. These findings indicate that regulatory compliance alone may not capture the health risks of pesticide mixtures. Strengthened monitoring, improved traceability, and cumulative risk approaches are needed to protect consumer health and support evidence-based regulation.

## 1. Introduction

Brazil ranks among the world’s leading agricultural producers and exporters, playing a crucial role in both national food security and the economy [1,2]. Benefiting from favorable climatic conditions and extensive agricultural land, the country has developed productive farming systems that rely on pesticide use to control pests and diseases and to maintain crop yields [1,2,3,4,5]. Although pesticides contribute to agricultural productivity, their extensive use has raised concerns regarding food safety, environmental contamination, and potential adverse effects on human health [6,7,8].

These substances and their degradation products can contaminate environmental compartments and food products, causing soil degradation, water and air pollution and harm to terrestrial and aquatic ecosystems [9,10,11,12,13]. Once in the environment, they may enter the human body through contaminated food or water, increasing the risk of numerous health effects, including neurodegenerative diseases, respiratory disorders, metabolic dysfunctions, reproductive problems, and cancer [14,15,16,17].

Because pesticides are intentionally applied to agricultural crops, residues may remain in food products even when these compounds are used in accordance with established agricultural practices. Consequently, dietary intake has become one of the main exposure pathways for the general population, representing a continuous and largely unavoidable source of exposure throughout life. This concern is relevant because consumers are rarely exposed to a single pesticide residue; instead, they are often simultaneously exposed to multiple compounds originating from different agricultural practices and food sources [11,14,15,16,18].

The occurrence of pesticide residues in food has become a global public health concern, extending beyond major agricultural-producing countries [12]. Monitoring programs conducted in Europe, North America, Asia, and Latin America consistently report multiple pesticide residues in the same food commodity, indicating that consumers are routinely exposed to complex chemical mixtures rather than individual compounds [19]. This exposure pattern has raised concern because chemicals with similar or complementary toxicological properties may contribute to additive, cumulative, or synergistic effects that are not adequately captured by assessments based on individual compounds alone [11,18].

Despite this reality, regulatory frameworks and conventional food safety assessments remain focused on single-compound evaluations. Maximum residue limits (MRLs) are generally established for individual pesticides, whereas cumulative effects resulting from simultaneous exposure to multiple residues are often insufficiently addressed. Consequently, compliance with regulatory limits does not necessarily imply the absence of health risks associated with combined dietary exposure [8,20].

These challenges are relevant in countries with intensive agricultural production and high pesticide consumption, where the diversity of active ingredients increases the likelihood of simultaneous dietary exposure to multiple residues [21]. In this context, Brazil represents an important case study, given the scale of its agricultural sector and the extensive use of pesticides across a wide range of crops [12]. Furthermore, some active ingredients currently authorized for agricultural use in Brazil remain restricted or banned in other jurisdictions because of concerns regarding environmental and human health effects, highlighting regulatory divergences and the need for country-specific evidence to support food safety policies and risk management strategies [18,21,22].

In Brazil, the Pesticide Residue Analysis Program in Food (Programa de Análise de Resíduos de Agrotóxicos em Alimentos—PARA) routinely monitors pesticide residues in foods consumed by the population and has repeatedly identified samples that exceed MRLs or contain pesticides not authorized for specific crops [8,20]. These findings reinforce concerns about dietary exposure to pesticide residues and underscore the importance of ongoing monitoring.

Although regulatory monitoring programs provide valuable information on pesticide occurrence and compliance, few studies have integrated occurrence data with cumulative risk assessment approaches that account for simultaneous exposure to multiple pesticides that share common toxicological targets and biological effects [18,21,22]. Therefore, the present study evaluated the occurrence of residues of currently used pesticides, selected for their widespread use, toxicological relevance, and representativeness across chemical classes, in commonly consumed plant-based foods sold in Brazil; assessed compliance with current regulations; and characterized potential human health risks associated with dietary exposure. Emphasis was placed on cumulative risk assessment using toxicological grouping approaches, providing additional insight into the relevance of pesticide mixtures for food safety and public health.

## 2. Material and Methods

### 2.1. Chemicals and Reagents

High-purity deionized water with a resistivity of 18.2 MΩ·cm was obtained from a Milli-Q system (Millipore RiOs-DI^®^, Bedford, MA, USA). Acetonitrile and ethyl acetate (HPLC grade) and acetic acid (purity ≥ 99%) were purchased from Sigma-Aldrich^®^ (St. Louis, MO, USA). Anhydrous magnesium sulfate (MgSO_4_) and sodium acetate (C_2_H_3_NaO_2_), used to optimize analyte purification and concentration, were supplied by Merck^®^ (Darmstadt, Germany). Dispersive solid-phase extraction (*d*-SPE) was performed using 150 mg of MgSO_4_, 50 mg of primary–secondary amine (PSA), and 50 mg of C18, all obtained from Waters^®^ (Milford, MA, USA). Final cleanup was carried out using Oasis^®^ Prime HLB (3 cc, 60 mg) solid-phase extraction (SPE) cartridges (also from Waters^®^).

**Figure 1:**
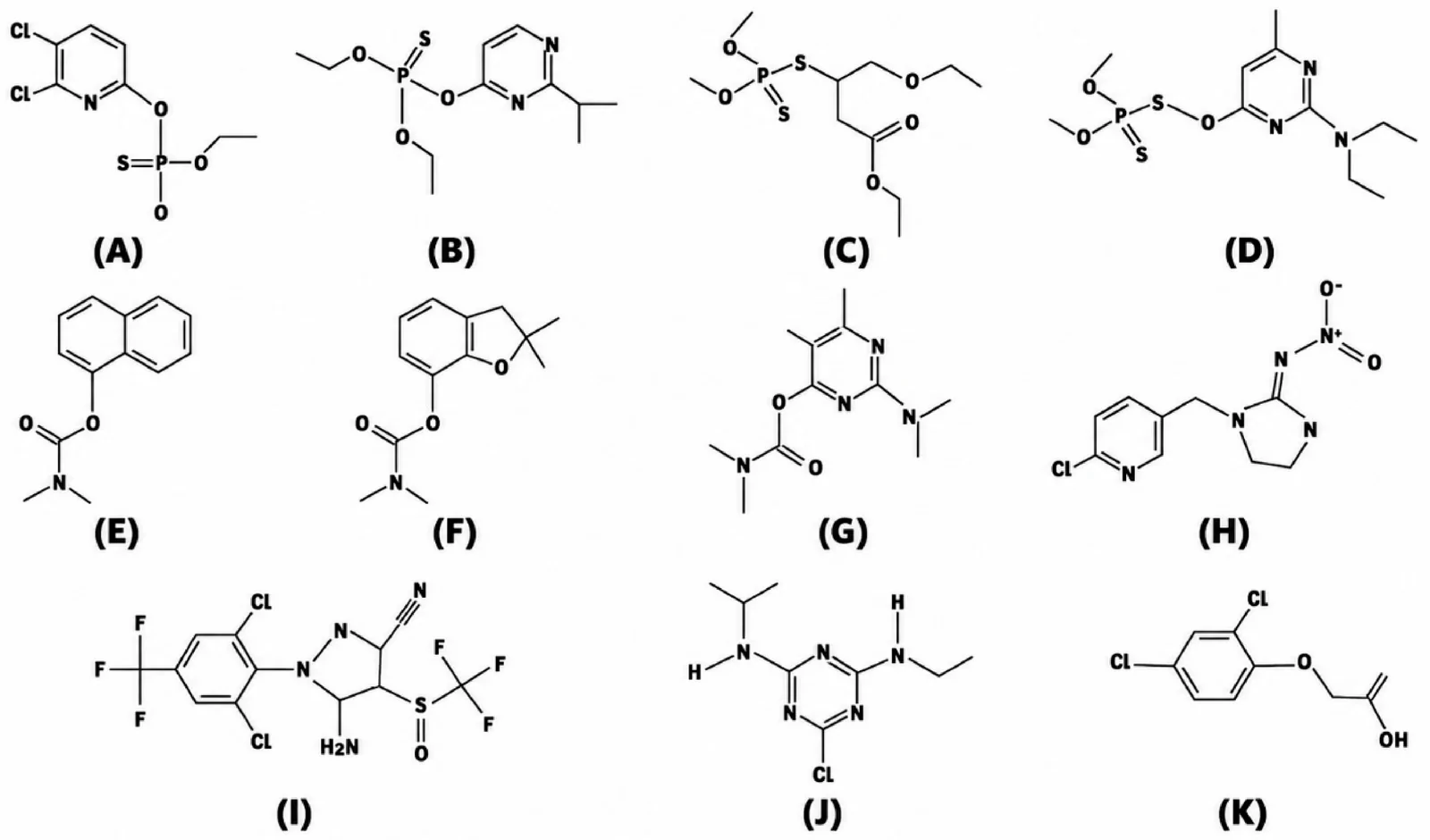
Pesticides included in this study: Chlorpyrifos (**A**), Diazinone (**B**), Malathion (**C**), Pirimifos (**D**), Carbaryl (**E**), Carbofuran (**F**), Pirimicarb (**G**), Imidacloprid (**H**), Fipronil (**I**), Atrazine (**J**), 2,4-dichlorophenoxyacetic acid (2,4-D) (**K**).

Eleven pesticide analytical standards, including 2,4-dichlorophenoxyacetic acid (2,4-D), atrazine, carbofuran, carbaryl, pirimicarb, malathion, diazinon, pirimiphos, chlorpyrifos, fipronil, and imidacloprid, were used for calibration and quantification. All were purchased from Sigma-Aldrich^®^ and are shown in Figure 1. The deuterium-labeled standards, carbofuran-*d*3 and imidacloprid-*d*4 (Sigma-Aldrich^®^), served as internal standards (ISTD). Individual stock solutions and working dilutions of native and internal standards were prepared in acetonitrile (ACN) and stored at −20 °C, protected from light, to preserve analyte stability until analysis.

### 2.2. Sample Collection

A total of 60 plant-based food samples, corresponding to 20 commonly consumed fruits, vegetables, and root crops (*n* = 3 per commodity), were purchased from supermarkets and open-air markets in southeastern Brazil. The commodities comprised orange, strawberry, grape, papaya, passion fruit, apple, peach, pear, mango, guava, tomato, carrot, sweet potato, sweet pepper, cucumber, kale, spinach, cassava, zucchini, and eggplant.

Each sample was individually homogenized to obtain a representative matrix for analysis. Only the edible portion of each sample was used for extraction. The homogenized samples were transferred to labeled 50 mL polypropylene centrifuge tubes and stored at −20 °C until sample preparation and instrumental analysis to preserve analyte stability.

### 2.3. Sample Preparation

**Figure 2:**
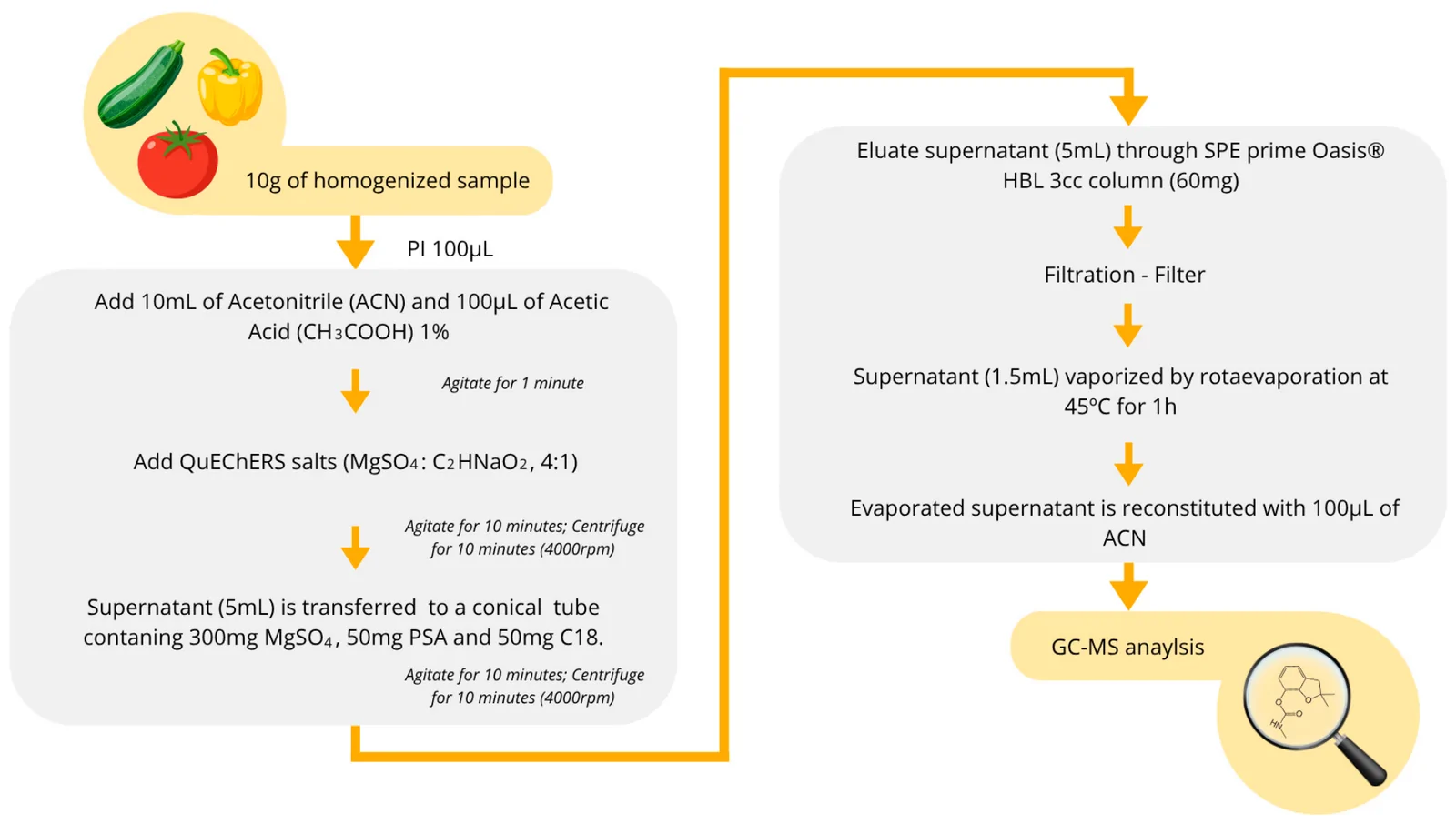
Sample preparation methodology, including the QuEChERS and SPE steps.

The sample preparation method for determining pesticides in plant-based food matrices with high water content was previously developed and validated by Prestes et al. (2009) and Souza et al. (2014), combining QuEChERS (Quick, Easy, Cheap, Effective, Rugged, and Safe) and solid-phase extraction (SPE) for efficient, broad-range extraction [23,24]. The workflow is summarized in Figure 2. Briefly, 10 g of the sample was fortified with an internal standard mixture solution of carbofuran-*d*3 and imidacloprid-*d*4 (at a final concentration of 5 mg kg^−1^). Acetonitrile (10 mL) and 1% *v*/*v* acetic acid (100 µL) were then added, and the mixture was vortexed for 1 min. In the salting-out step of QuEChERS, MgSO_4_ and C_2_H_5_NaO_2_ were added, then the mixture was shaken and centrifuged for 10 min. The supernatant was transferred to a conical tube containing d-SPE sorbents (MgSO_4_, PSA, and C18), compounds used to remove co-extracted matrix components that could interfere with chromatographic analysis. After vortexing and centrifugation, the extract was loaded onto an Oasis^®^ Prime HLB cartridge (3 cc, 60 mg). The cartridge was washed with 2 mL of Milli-Q water, and the analytes were eluted with 1.5 mL of ethyl acetate and filtered through a polytetrafluoroethylene (PTFE) membrane. The eluate was concentrated, and the dry residue was reconstituted in 100 µL of ACN for instrumental analysis. 

### 2.4. Instrumental Analysis

The eleven Current Use Pesticides (CUPs) were analyzed sequentially using a gas chromatograph coupled to a single quadrupole mass spectrometer (GC-MS; Thermo Fisher Scientific^®^, Waltham, MA, USA). The instrumental method was based on Komatsu et al. (2004), Rissato et al. (2006), and Prates et al. (2011), and adjusted to the physicochemical characteristics of the target analytes [23,25,26]. A 2 µL volume was injected at 280 °C in splitless mode, with the split valve opening after 1 min to minimize analyte dilution and improve sensitivity. High-purity helium (99.9999%) was used as the carrier gas at a constant flow rate of 1.2 mL min^−1^. Chromatographic separation was performed on an FS-CAP SLB-5MS column from Sigma-Aldrich^®^ (30 m × 0.25 mm × 0.25 μm; 5% diphenyl polysiloxane, 95% dimethyl polysiloxane). The oven temperature was initially held at 80 °C for 1 min, then ramped at 40 °C min^−1^ to 280 °C and held for 12 min, giving a total run time of 18 min. The transfer line and ion source temperatures were set to 280 °C and 240 °C, respectively. Data were acquired in Selected Ion Monitoring (SIM) mode. The m/z values and retention times of each compound are listed in Table S1 (Supplementary Information).

### 2.5. Quality Control and Method Validation

Organic plant-based food produced without pesticide application was obtained from local markets and homogenized into a single pooled matrix to encompass potential interferents and matrix characteristics across commodities. This pool served as a blank for method development and was analyzed in multiple replicates to assess selectivity. The method was validated in accordance with the SANTE/11312/2021 guideline on analytical quality control and method validation for pesticide residues in food and feed [27]. The performance parameters evaluated were matrix effect, selectivity, limits of detection (LOD) and quantification (LOQ), linearity, precision, and accuracy. The results are summarized in Table S2 (Supplementary Information). Calibration curves were constructed by weighted least-squares linear regression of the analyte-to-internal-standard peak area ratios across seven concentration levels, analyzed in triplicate. Linearity was satisfactory over the range of 0.001–10 mg kg^−1^ for all analytes with quality control levels set at 0.05, 4, and 8 mg kg^−1^. Sampling quality control included systematic documentation of sample provenance. Sample representativeness was ensured by collecting different groups of fruits and vegetables from the state of São Paulo, Brazil, and systematically documenting sample provenance. Method blanks (solvent only) were processed with each analytical batch. Samples were stored at −20 °C and analyzed within 72 h after extraction. All sample handling was performed in a clean laboratory environment. The analytical sequence included initial calibration verification, method blanks, laboratory control samples, and sample duplicates to ensure data quality and method performance throughout the study. 

### 2.6. Health Risk Characterization

Non-carcinogenic (HQ) and carcinogenic (CRisk) risks were estimated to assess potential human health impacts. Risk calculations incorporated standardized consumption data from the Brazilian Family Budget Survey (Pesquisa de Orçamentos Familiares—POF), conducted by the Brazilian Institute of Geography and Statistics [28]. A reference adult body weight of 70 kg was adopted, and chemical stability was assumed during food preparation and consumption. Risk metrics were calculated according to Souza et al. (2021) [29], using Equations (1)–(4):

(1) $$EDI\;=\frac{\left(C\;\cdot \;IR\right)}{BW}$$

(2) $$HQ=\frac{EDI}{RfD}$$

(3) HI=sum HQ

(4) CRisk=EDI · oSF 

where:

- EDI: Estimated Daily Intake
- C: Average pesticide concentration in food (mg kg^−1^)
- IR: Ingestion Rate (kg day^−1^)
- BW: Body Weight (70 kg)
- HQ: Hazard Quotient (dimensionless)
- RfD: Reference oral dose (mg/kg/day)
- HI: Hazard Index (dimensionless)
- CRisk: Cancer risk (dimensionless)
- oSF: Oral Slope factor (mg/kg/day)^−1^

Because a Reference Dose (RfD) for pirimiphos-ethyl was not available in any Regional Screening Level table or official source [19,30], a read-across strategy was applied, adopting the pirimiphos-methyl value, set at 7.30 ×·10^−3^ mg/kg/day, based on structural similarity [19,30,31]. Read-across predicts toxicological properties of data-poor compounds from structurally related compounds [32]. Although it introduces uncertainty, it still provides informative endpoints.

Risk assessment of dietary exposure to chemical mixtures requires grouping compounds according to their toxicological properties. However, compounds with similar physicochemical traits, often grouped together during the development of extraction and instrumental methods, may exhibit distinct systemic effects and mechanisms of action. Accordingly, the EFSA, “Guidance Document on Scientific criteria for grouping chemicals into assessment groups for human risk assessment of combined exposure to multiple chemicals,” establishes criteria for categorizing compounds by toxicological profile [33].

The assessment was based on EFSA cumulative assessment groups (CAGs), which group compounds by shared properties, such as target organ (CAG level 1), phenomenological effect on the target organ (CAG level 2), mode of action behind a specific effect (CAG level 3), and the mechanism behind the effect (CAG level 4) [34,35,36]. The present study focuses on CAG level 2, divided into nervous and endocrine effects. Within the nervous category, several pesticides showed evidence of functional effects on the motor, sensory, and autonomic divisions, including blocking GABA receptors.

Average pesticide concentrations determined by arithmetic mean of the replicates for each commodity were used to define exposure scenarios, providing conservative estimates of human health risks. For each commodity, three replicates were considered: when one or two replicates were below the LOD, a value of LOD/√2 was assigned to those replicates (medium-bound scenario); when all three replicates were below the LOD, the commodity was excluded from the risk assessment [37].

Risk thresholds followed US EPA (2022) guidance, which defines HI > 1.0 as indicative of potential chronic systemic effects, and CRisk > 1 × 10^−5^ as indicative of elevated lifetime cancer risk [38]. Complementary interpretation followed Center for Disease Control and Prevention, with CRisk values between 1 × 10^−6^ and 1 × 10^−4^ considered tolerable. The parameters used in the risk assessment are listed in Table S3 (Supplementary Information) [39].

### 2.7. Statistical Analyses

Statistical analyses were performed using GraphPad Prism (version 8; GraphPad Software, San Diego, CA, USA) and Microsoft Excel (Microsoft Corp., Redmond, WA, USA). Descriptive statistics were used to summarize the data, and mean pesticide concentrations were calculated for each commodity. In addition, calculations related to the validation of analytical methodology, including accuracy, precision, linearity, limits of detection (LOD), and limits of quantification (LOQ), were performed using these software packages.

## 3. Results and Discussion

### 3.1. Occurrence of Pesticide Residues in Plant-Based Food

**Figure 3:**
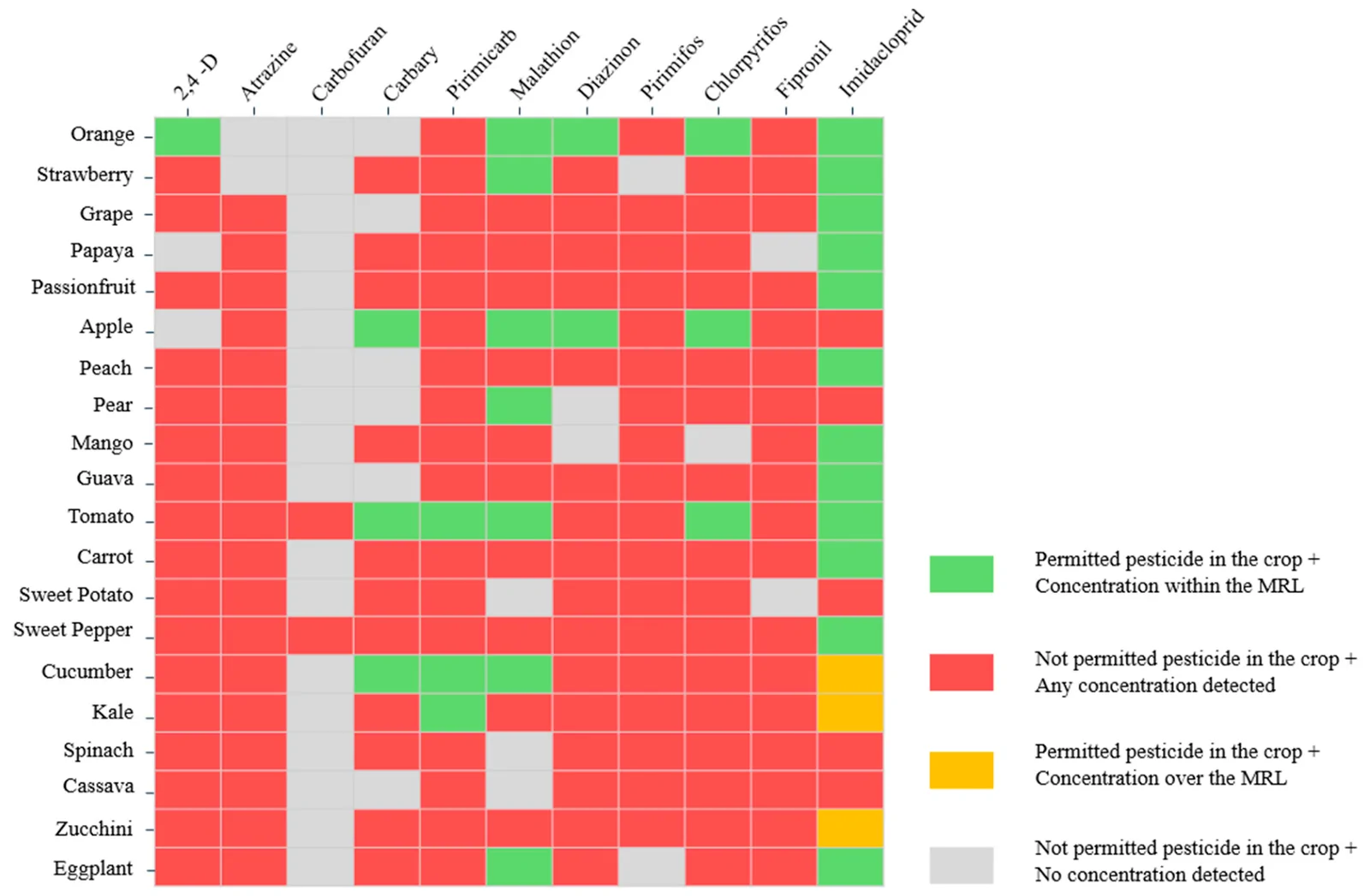
Heatmap combining the concentration of pesticides in the analyzed samples and their permission within Brazilian legislation.

The occurrence and concentrations of CUP residues in the analyzed commodities are presented in Table 1. The arithmetic mean concentrations were calculated from three independent samples per commodity, providing an overview of residue profiles across fruits, vegetables, and root crops commonly consumed in Brazil. To support a broader assessment of occurrence patterns and regulatory status, the results are also presented as a heatmap (Figure 3) that integrates the authorization status of each pesticide-crop combination and compliance with MRLs. This representation allows the identification of commodities and active ingredients most frequently associated with regulatory non-compliance (Table S4, Supplementary Information).

Figure 3 provides an integrated view of pesticide occurrence, regulatory compliance, and potential risk across crops, revealing heterogeneous patterns among the evaluated commodities. Green cells (permitted and below MRL) denote regulatory compliance and acceptable exposure, indicating that, for these pesticide-crop combinations, agricultural practices align with established safety standards, as seen in a few crops that use 2,4-D, carbaryl, pirimicarb, malathion, and imidacloprid. Yellow cells (permitted but above MRL) denote authorized pesticides whose residues exceeded regulatory thresholds. These cases are relevant from a food safety perspective, as they reflect misuse, such as over-application, inadequate pre-harvest intervals, or environmental accumulation [40,41,42]. This pattern was restricted to cucumber, kale, and zucchini, with imidacloprid concentrations exceeding the MRL, reaching 4.0 mg kg^−1^ in cucumber and kale and 3.5 mg kg^−1^ in zucchini.

More concerning are the red cells (not permitted but detected), which constitute clear regulatory violations. The presence of unauthorized pesticides in a given crop may result from off-label use, whether intentional or inadvertent, or contamination during processing and distribution. Strictly speaking, when an active ingredient is not approved for a particular crop, its presence in that food matrix should be classified as contamination rather than a pesticide residue; for simplicity, however, the term “pesticide residue” is used throughout this study to refer to all such cases. The recurrence of this pattern across commodities, rather than isolated events, suggests systemic issues in pesticide management, including off-label application, insufficient adherence to regulatory guidelines, or cross-contamination along the production chain. Several pathways may explain the presence of these residues in crops for which they are not authorized, including spray drift from neighboring fields, contamination of irrigation water, persistence of compounds in soil from previous cropping cycles, and equipment cross-contamination during mixing or application [12,43,44,45,46].

From a food safety perspective, this widespread occurrence raises concerns not only about regulatory compliance but also about cumulative and combined exposure, given the co-occurrence of multiple residues within single samples. These findings align with PARA reports, which frequently detected unauthorized pesticide residues in food matrices, accounting for about 26% of all analyses in the most recent monitoring cycle. Although currently used pesticides are generally less persistent than legacy compounds, they can still undergo long-range transport and environmental cycling, depending on the mode of active-ingredient release, the application method, and the stability of the commercial formulation [44,45]. Such environmental dispersion across production areas can lead to the presence of non-authorized residues in crops and is associated with the pollution of water, soil, and air [12,47,48].

Gray cells (not permitted and not detected) reflect expected regulatory compliance, indicating either no use or effective control measures that prevent contamination. The predominance of gray and green cells in certain crops suggests better compliance and possibly more controlled production systems. Carbofuran, in particular, should not be detected in any crop, as it has been banned in Brazil since 2017. Nevertheless, it was still identified in certain commodities, such as tomato and sweet pepper, at concentrations of 0.017 and 0.023 mg kg^−1^, respectively. Although these levels are low, their presence may suggest environmental persistence of the active ingredient or residual contamination from past applications [49,50].

Some pesticides were detected across multiple commodities, suggesting either widespread use or environmental persistence. Likewise, certain crops showed a higher frequency of non-compliant results, which may be linked to their agronomic characteristics, pest pressure, or differences in regulatory oversight. From a public health and regulatory standpoint, the detection of unauthorized pesticides underscores the need for improved traceability systems and stricter enforcement of pesticide regulations, particularly where regulatory frameworks are applied inconsistently.

### 3.2. Regulatory Compliance Assessment

**Figure 4:**
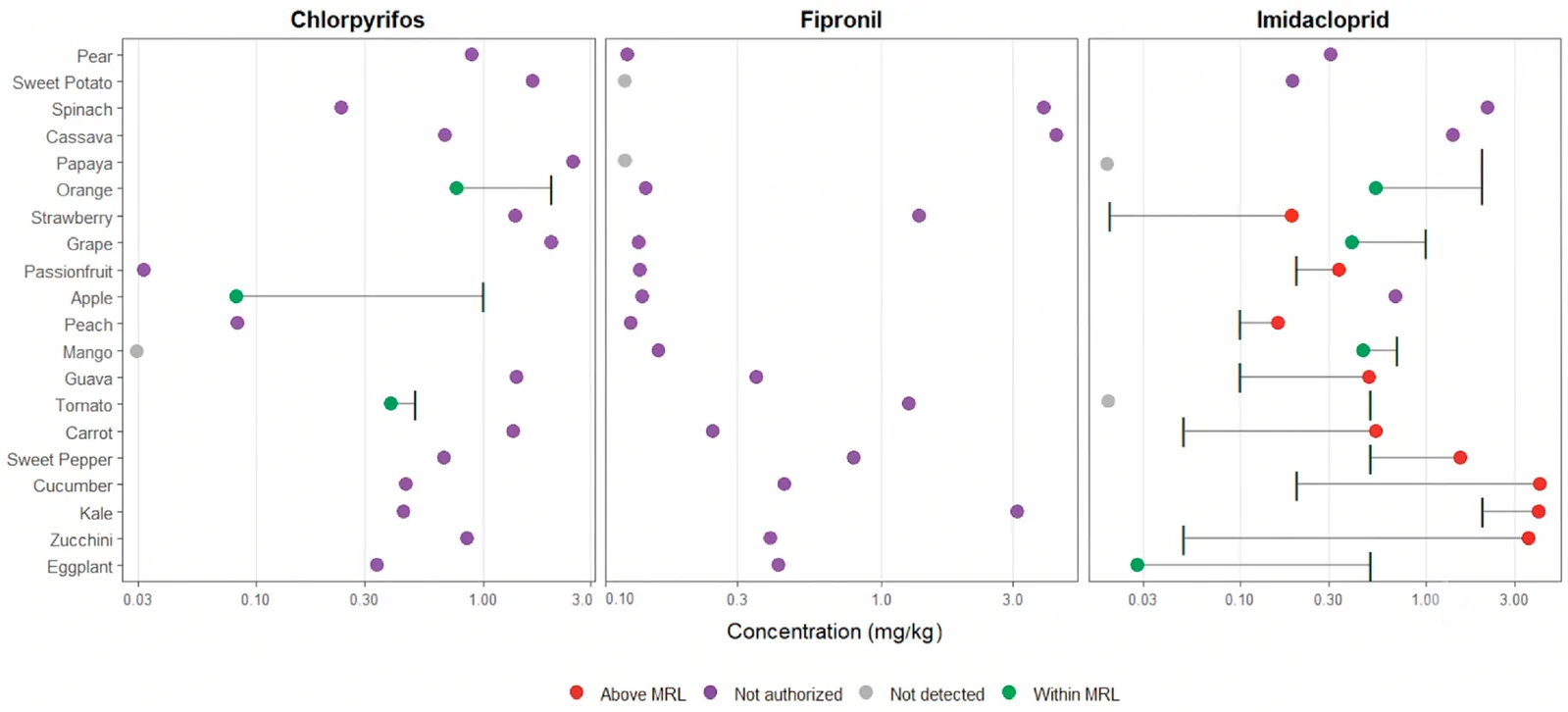
Comparison between the measured concentrations and the corresponding Brazilian maximum residue limits (MRLs) for imidacloprid, chlorpyrifos, and fipronil. Colored circles represent the measured concentrations in individual samples, while black vertical markers indicate the crop-specific Brazilian MRLs. Grey connecting lines facilitate the comparison between each measured concentration and its respective MRL. Point colors indicate the regulatory status of each detection: residues within the established MRL (green), residues exceeding the MRL (red), and residues detected in crops without an established Brazilian MRL (purple). The x-axis is presented on a logarithmic scale to accommodate the wide range of concentration and MRL values.

In the 2023 cycle, PARA analyzed 3294 fresh food samples, including fruits, vegetables, and widely consumed root crops. Of these, 226 samples (6.9%) contained pesticide residues above the MRLs established by regulation [8,20]. These findings are consistent with our results, which revealed that several pesticides, particularly chlorpyrifos, imidacloprid, fipronil and others, exhibited inconsistencies with the current Brazilian legislation. The main cases are discussed below and graphically compared with the applicable crop-specific MRLs in Figure 4.

To facilitate the interpretation of the analytical results, the concentrations of the three most frequently detected pesticides were graphically compared with their respective crop-specific Brazilian MRLs (Figure 4). The logarithmic scale was adopted because both residue concentrations and regulatory limits span approximately two orders of magnitude, allowing low and high values to be displayed simultaneously without compromising readability.

Pirimiphos-methyl is an organophosphorus insecticide used to control a wide range of insect pests [51]. Although organophosphorus compounds are primarily associated with acute intoxication through inhibition of acetylcholinesterase, growing evidence indicates that these substances may also cause compound-specific chronic effects, including delayed polyneuropathy, immunotoxicity, carcinogenesis, and endocrine, developmental, and reproductive toxicity [51,52,53].

Pirimiphos-methyl was among the active ingredients associated with recurrent noncompliance, primarily because it was detected in crops where its use is not authorized. Its presence in widely consumed matrices contributed to the classification of several samples as unsatisfactory. The off-label use of pirimiphos-methyl in foods intended for human consumption raises concerns about cumulative exposure, especially when considered together with other organophosphorus insecticides frequently detected in the PARA monitoring program, such as chlorpyrifos and malathion. 

Chlorpyrifos is a broad-spectrum insecticide used in agricultural systems and residential settings [54]. Its use is widespread in both developed and developing countries, across a wide variety of crops [55]. It has been linked to several toxicological effects in humans, including endocrine disruption and reproductive, developmental, and neurological toxicity [56,57]. Chlorpyrifos was one of the most problematic pesticides identified in this study. Residues were detected in several commodities, including strawberry, grape, guava, carrot, and sweet potato, at concentrations ranging from 0.032 to 1.995 mg kg^−1^. In most cases, detections occurred in crops for which chlorpyrifos is not authorized, resulting in frequent regulatory non-compliance and raising concerns about the effectiveness of current control measures. A similar pattern has been reported by PARA, which detected chlorpyrifos in 275 food samples nationwide, more than 85% of them classified as irregular under current regulations [20]. Chlorpyrifos was, in fact, the active ingredient associated with the largest number of non-compliant results in the program. The widespread occurrence of irregular chlorpyrifos residues may reflect a combination of factors, including unauthorized use, spray drift, environmental persistence, contamination of water and soil, and cross-contamination during agricultural operations [58,59].

Malathion, regarded as an alternative to chlorpyrifos, is widely used under the assumption that it is less toxic than other organophosphates [60,61,62]. Despite this relative safety, malathion exposure has been associated with increased cancer risk, in line with its genotoxic and carcinogenic profile, with neurotoxicity through cholinergic and non-cholinergic mechanisms, and with hepatotoxicity and nephrotoxicity [60,62]. Malathion residues were detected in 17 of 20 commodities (85%) at concentrations ranging from 0.012 to 0.297 mg kg^−1^. Although it is among the most extensively marketed pesticide active ingredients in Brazil, most malathion detections, both in the present study and in PARA (85.7%), complied with current regulatory limits [18,20]. These results suggest that, unlike chlorpyrifos, malathion is generally associated with lower regulatory non-compliance. Differences between the two datasets should be interpreted with caution, as only a limited number of commodities overlapped. Moreover, PARA data indicate that compliant malathion detections were largely concentrated in orange, which may overrepresent its apparent national frequency. In the present study, residues below the MRL were found in only seven commodities (orange, strawberry, apple, pear, tomato, cucumber, and eggplant), indicating a more restricted distribution across crop types.

Concerns about neonicotinoids have implications for human, animal, and environmental health [63,64]. Imidacloprid is a potent insecticide that acts as an agonist at nicotinic acetylcholine receptors, causing persistent receptor activation, neuronal hyperexcitation, and ultimately insect paralysis and death; it is used to control a wide variety of pests in agriculture [65,66]. Owing to its extensive use, its toxicity to non-target organisms has been frequently reported [67,68]. In particular, these non-target effects have highlighted the risks to pollinators, such as bees, whose ecological role supports ecosystem functioning and biodiversity [64,67,69].

Imidacloprid was among the most frequently detected insecticides in PARA, accounting for 16.7% (n = 550) of all detections, together with tebuconazole and difenoconazole [20]. Its frequent occurrence is likely related to its extensive agricultural use, as the compound is currently authorized for approximately 60 crops in Brazil [70]. Despite this high detection frequency, only 13.4% (n = 74) of the imidacloprid findings reported by PARA were classified as non-compliant, more than half of which exceeded the established MRLs.

A different pattern was observed in the present study, in which imidacloprid was among the pesticides most frequently associated with regulatory irregularities. Overall, 31.7% of the analyzed samples showed non-compliant detections, including 13.4% with concentrations exceeding the corresponding MRLs and 18.3% involving crops for which the active ingredient is not authorized. The highest concentrations occurred in cucumber, kale, and zucchini; zucchini showed the most pronounced exceedance, reaching 3.57 mg kg^−1^, more than 70-fold the established MRL (0.05 mg kg^−1^). Among crops where imidacloprid is not permitted—apple, pear, sweet potato, spinach, and cassava—residue concentrations ranged from 0.19 to 2.16 mg kg^−1^.

These results suggest that the occurrence of pesticide residues cannot be explained solely by authorized agricultural use and may reflect a combination of factors, including misuse, off-label application, environmental dispersion, and cross-contamination along the production chain. The detection of residues in crops for which the active ingredient is not authorized highlights persistent challenges in pesticide management and regulatory enforcement. Moreover, establishing MRLs and restrictions on pesticide use do not, in themselves, prevent the occurrence of residues in food, underscoring the need for continuous monitoring, effective traceability systems, and strengthened surveillance programs.

Among the pesticides identified in the present study, fipronil warrants particular attention due to its prevalence and toxicological relevance. Fipronil is a phenylpyrazole insecticide widely used in agriculture, veterinary medicine, and public health programs [71]. It acts as a non-competitive antagonist of γ-aminobutyric acid (GABA)-gated chloride channels, blocking chloride influx and leading to neuronal hyperexcitation in target insects. Its higher affinity for insect over mammalian GABA receptors accounts for its selective toxicity. Although effective for pest control, its extensive use has raised concerns about environmental contamination and potential adverse effects on non-target organisms [71,72,73]. Experimental and toxicological studies have demonstrated that fipronil may induce a variety of adverse outcomes, including cytotoxic, reproductive, hepatotoxic, neurotoxic, and neurodegenerative effects in both vertebrate and invertebrate species [73,74,75,76]. Owing to its long half-life and extensive use, fipronil can reach non-target organisms through indirect exposure pathways, including ingestion of contaminated water, pollen, and produce [71,77]. This environmental persistence poses health and ecological hazards, particularly given its high toxicity to pollinators, such as bees, and to aquatic organisms; consequently, it has been banned or restricted in several countries [78,79].

Fipronil was among the active ingredients most frequently associated with irregularities, predominantly as an unauthorized residue in specific crops. These cases mainly reflected its detection in crops for which its use is not authorized, indicating shortcomings in compliance with registration requirements and good agricultural practices. From a toxicological perspective, although most detections did not exceed the acute reference dose limits, the presence of fipronil as a non-authorized residue raises concerns about chronic exposure and the effectiveness of regulatory oversight and enforcement of this insecticide.

In addition, widely consumed crops such as tomato, apple, mango, and strawberry contained simultaneous residues of multiple pesticides above their legal limits, reinforcing concerns about combined dietary exposures. In contrast, active ingredients such as atrazine and imidacloprid showed a higher frequency of compliance with current legislation, although isolated exceedances were still observed. 

This scenario reveals not only shortcomings in the implementation of good agricultural practices but also limitations in the regulatory framework in addressing combined exposures, particularly for foods that constitute a substantial part of the population’s diet. Overall, these findings underscore the need to strengthen monitoring programs, traceability measures, and to periodically re-evaluate active ingredients that consistently show non-compliance.

### 3.3. Human Health Risk Characterization

Risk characterization integrates hazard identification, dose-response assessment, and exposure evaluation to estimate the likelihood of adverse effects from pesticide residues in an individual or population under given exposure conditions. In dietary risk characterization, a potential risk exists when exposure exceeds established toxicological reference values [80].

Among the 11 pesticides evaluated, only atrazine has an available Slope Factor, allowing the quantitative estimation of carcinogenic risk (Table 2). The absence of a slope factor for the remaining compounds should not be taken as evidence of no carcinogenic potential. In fact, some of the pesticides detected in this study, such as 2,4-D, carbaryl, diazinon, and malathion, have been classified by international agencies as having possible or probable carcinogenic effects [81].

Recent studies suggest that exposure to pesticide mixtures may contribute to carcinogenic risk through cumulative or synergetic mechanisms [82,83]. A key limitation in cancer risk characterization is the scarcity of toxicological data and quantitative estimates of cancer potency for many pesticides, which may lead to an underestimation of the carcinogenic risk associated with mixtures.

Based on the available data, dietary exposure to atrazine does not appear to pose a significant risk, with the highest estimated value observed in sweet pepper samples (3.85 × 10^−7^). However, this estimate considers a single food group, whereas individuals consume a variety of foods daily, potentially leading to cumulative atrazine exposure from multiple dietary sources. When all analyzed food commodities consumed by the Brazilian population were considered, the estimated total lifetime cancer risk associated with dietary atrazine exposure was 3.28 × 10^−6^. Simultaneous exposure to multiple pesticide residues may also occur, increasing the overall toxicological burden [84]. For these reasons, the use of a 1 × 10^−5^ threshold to define cancer risk may be questioned, as it can be readily exceeded, leading to underestimation of the present findings.

The key indicators of the specific effects observed for each CAG level 2 are presented in Table S5. Some compounds did not show specific effects, but their modes of action were identified in the EFSA scientific report and therefore considered here. Only pesticides confirmed by in vivo oral-exposure studies (diet, gavage, or capsules) were included; those not cited in the report were excluded. For the nervous system, the 2019 EFSA report replaced the CAG level 2 ‘neurochemical endpoints’ with ‘brain and/or erythrocyte acetylcholinesterase inhibition’ used in 2013, as none of the 11 pesticides showed relevant effects; this level was omitted. For thyroid effects, only the hypothyroidism CAG level 2 was considered, since “C-cell hypertrophy, hyperplasia, and neoplasia” relates more to carcinogenic risk, and atrazine was not cited in the report.

Regarding the results in Table 3, the standout samples were strawberry and cassava, which showed considerable HI values for functional effects on the motor, sensory, and autonomic divisions. For the latter, cassava also showed a pronounced effect on the thyroid. These indicators were nearly 3 times the HI threshold and were strongly influenced by the high detected fipronil levels. Although an MRL for this pesticide in any of the 11 food matrices is not available in Brazilian legislation, using the MRL of 0.05 mg kg^−1^ based on Regulation (EU) 2024/347, the average concentration found in cassava was 4.31 mg kg^−1^, showing a discrepancy of 86.2 times higher [85]. The HI ranged between 2.77 and 3.16 for cassava, with fipronil HQ representing 88–99% of the sum.

The same pattern holds for strawberries and spinach. Although the fruit results for HI are slightly above 1.00 and the vegetable results sometimes deviate slightly below the criterion value, fipronil is the major contributor to the calculated hazard in both cases, at 83.04% for strawberry samples and 94.44% for spinach. Notably, fipronil levels are much higher than expected for food safety, resulting in these HI values, even though it had the lowest RfD among all 11 pesticides analyzed.

Risk assessment results can provide only a limited view of the actual presence and concentrations of pesticides. For some commodities, when a pesticide is considered in isolation, one replicate may show a concentration below the LOD, while another may be a few times higher for the same parameter. This introduces uncertainty into the analysis, since one consumer may be exposed to batches with the highest values, while another consumer may be exposed to safer products, contradicting the medium-bound scenario used in this assessment. Furthermore, some of the analyzed pesticides, when they undergo biotransformation during absorption, distribution, metabolism, and excretion (ADME), can produce intermediate compounds and degradation products that also exhibit toxicological effects and, consequently, can alter the HQs and final HIs for compound mixtures in risk assessment.

It is also important to note that the absence of an RfD for pirimiphos-ethyl in non-carcinogenic risk assessment, as well as the absence of an oSF for 2,4-D and imidacloprid in carcinogenic risk assessment, despite their presence in the EFSA scientific report for endocrine CAGs, leads to limited conclusions about the real risks associated with the consumption of Brazilian commodities. Furthermore, the groups of pesticides and foods investigated in this study do not include all compounds and matrices reported in PARA; additional investigations could complement these findings. Moreover, incorporating multiplicative factors such as bioaccessibility and bioavailability ratios for ingestion exposure into the risk assessment equations could yield more realistic estimates of the pesticide concentrations and risks to which a consumer may be exposed.

Another limitation of this study is the relatively small sample size; only 60 plant-based food samples were included, which may not fully capture the variability of products available on the market. In addition, exposure and risk assessments were based on mean concentration values, providing an average estimate that may not reflect high-end exposure scenarios. Therefore, caution should be exercised when extrapolating these findings to broader populations or using them in a regulatory context, and further studies including larger datasets and probabilistic approaches are recommended.

## 4. Conclusions

This study provides a comprehensive assessment of pesticide residue occurrence, regulatory compliance, and potential dietary health risks associated with commonly consumed plant-based foods in Brazil. Although most detected residues complied with current regulatory standards, and the estimated carcinogenic risk associated with atrazine remained below accepted thresholds, several commodities contained residues exceeding MRLs or active ingredients not authorized for the corresponding crops. These findings highlight persistent challenges related to pesticide management, regulatory compliance, and food safety surveillance.

Importantly, the cumulative risk assessment revealed potential health concerns that would not have been identified through conventional single-compound evaluations alone. Cassava, strawberry, and spinach presented hazard index (HI) values above the acceptable threshold, with cassava showing the highest cumulative risk, largely driven by elevated fipronil concentrations. These results indicate that compliance-based assessments may underestimate health risks when simultaneous exposure to multiple pesticide residues is not considered.

The detection of unauthorized pesticides and the occurrence of cumulative risks reinforce the need to incorporate mixture-based approaches into food safety evaluations. Current regulatory frameworks are designed primarily around individual compounds, whereas consumers are routinely exposed to complex combinations of pesticide residues through their diet. Consequently, cumulative risk assessment should be progressively integrated into monitoring programs and regulatory decision-making.

Some limitations should be acknowledged. Samples were collected from a single geographic region and may not fully represent the diversity of agricultural practices, environmental conditions, and pesticide use patterns across Brazil. In addition, toxicological reference values remain unavailable for several active ingredients, limiting a more comprehensive characterization of cumulative and carcinogenic risks. Uncertainties related to bioavailability, bioaccessibility, degradation products, and interactions among pesticide mixtures may also contribute to the underestimation of dietary health risks.

Despite these limitations, the present study demonstrates the value of integrating occurrence data, regulatory compliance assessments, and cumulative risk characterization to provide a more realistic evaluation of dietary pesticide exposure. Future studies should expand the range of commodities, geographic regions, and active ingredients analyzed, while incorporating refined exposure models and mixture toxicity approaches to better characterize real-world exposure scenarios and support evidence-based food safety policies.

## Figures and Tables

**Table 1:** Arithmetic mean concentrations of pesticides in fruits and vegetables (mg/kg).

	2,4-D	Atrazine	Carbofuran	Carbaryl	Pirimicarb	Malathion	Diazinon	Pirimiphos	Chlorpyrifos	Fipronil	Imidacloprid
Orange	<LOD *	<LOD	<LOD	<LOD	0.2482	0.0165	0.3896	0.3746	0.7692	0.1396	0.5404
Strawberry	0.0172	<LOD	<LOD	0.0389	0.1684	0.0671	<LOD	<LOD	1.3896	1.3752	0.1905
Grape	0.0708	0.0106	<LOD	<LOD	0.1658	0.0115	<LOD	0.0200	1.9956	0.1326	0.3994
Papaya	<LOD	<LOD	<LOD	<LOD	0.0425	0.0197	0.0416	<LOD	2.5041	<LOD	<LOD
Passionfruit	0.0346	0.0076	<LOD	0.0415	0.0288	0.0136	<LOD	0.1122	0.0318	0.1332	0.3435
Apple	<LOD	0.0008	<LOD	<LOD	0.3932	0.2432	0.6550	1.3823	0.0815	0.1354	0.6916
Peach	0.0423	0.0202	<LOD	<LOD	0.0490	0.0279	0.1969	0.3435	0.0822	0.1235	0.1593
Pear	0.0519	0.0015	<LOD	<LOD	0.1894	0.2966	<LOD	0.2194	0.8969	0.1195	0.3110
Mango	0.0080	0.0004	<LOD	0.0399	0.0648	0.0120	<LOD	0.6082	<LOD	0.1554	0.4634
Guava	0.0545	0.0048	<LOD	<LOD	0.0694	0.0298	0.1655	0.3380	1.4045	0.3516	0.4935
Tomato	0.0259	0.0042	0.0172	<LOD	0.1229	0.0463	0.0416	0.6632	0.3906	1.2642	<LOD
Carrot	0.1786	0.0082	<LOD	0.1952	0.0727	0.0393	2.6194	0.0778	1.3542	0.2455	0.5367
Sweet Potato	0.0378	0.0044	<LOD	0.1418	0.0326	<LOD	0.2511	0.0758	1.6599	<LOD	0.1919
Sweet Pepper	0.0351	0.0172	0.0227	0.3990	0.0187	0.1252	0.7038	0.0735	0.6750	0.7909	1.5266
Cucumber	0.0167	0.0008	<LOD	0.3923	<LOD	0.1717	0.7191	1.9421	0.4554	0.4429	4.0779
Kale	0.0277	0.0043	<LOD	0.4366	0.0745	0.2431	0.9501	0.0984	0.4478	3.1255	4.0153
Spinach	0.0400	0.0024	<LOD	0.4849	0.0470	<LOD	0.2453	0.3837	0.2380	3.8990	2.1577
Cassava	0.1260	0.0077	<LOD	<LOD	0.0854	<LOD	0.6938	0.9359	0.6797	4.3115	1.3952
Zucchini	0.0673	0.0057	<LOD	0.2919	0.2476	0.1186	0.3712	0.2482	0.8531	0.3958	3.5729
Eggplant	0.0247	0.0013	<LOD	0.6594	0.2642	0.2371	1.0316	<LOD	0.3425	0.4242	0.0279

* <LOD: Concentration below the limit of detection. Consistent with successive cycles of the Brazilian Pesticide Residue Analysis Program in Food (PARA), which frequently report the co-occurrence of multiple residues in the same sample, the present study confirms that consumers are commonly exposed to pesticide mixtures rather than isolated compounds [20]. Because regulatory assessments remain largely based on individual compounds, this pattern may underestimate the health risks of cumulative exposure, reinforcing the need for the broader risk-assessment approach adopted here [17,22].

**Table 2:** Cancer risk of each sample based on Atrazine exposure.

Food Sample	IR * (kg/day)	C ** (mg/kg)	EDI *** (mg/kg bw day)	CRisk
Orange	1.07 × 10^−2^	-	-	-
Strawberry	8.80 × 10^−3^	-	-	-
Grape	8.80 × 10^−3^	1.06 × 10^−2^	1.33 × 10^−6^	3.06 × 10^−7^
Papaya	6.20 × 10^−3^	1.73 × 10^−3^	1.53 × 10^−7^	3.52 × 10^−8^
Passionfruit	8.80 × 10^−3^	7.58 × 10^−3^	9.53 × 10^−7^	2.19 × 10^−7^
Apple	9.20 × 10^−3^	7.57 × 10^−4^	9.95 × 10^−8^	2.29 × 10^−8^
Peach	8.80 × 10^−3^	2.02 × 10^−2^	2.54 × 10^−6^	5.84 × 10^−7^
Pear	8.80 × 10^−3^	1.51 × 10^−3^	1.90 × 10^−7^	4.37 × 10^−8^
Mango	3.50 × 10^−3^	2.82 × 10^−2^	1.41 × 10^−6^	3.24 × 10^−7^
Guava	8.80 × 10^−3^	4.79 × 10^−3^	6.02 × 10^−7^	1.39 × 10^−7^
Tomato	4.20 × 10^−3^	4.89 × 10^−2^	2.93 × 10^−6^	6.75 × 10^−7^
Carrot	9.00 × 10^−4^	8.20 × 10^−3^	1.05 × 10^−7^	2.43 × 10^−8^
Sweet Potato	6.60 × 10^−3^	4.40 × 10^−3^	4.14 × 10^−7^	9.35 × 10^−8^
Sweet Pepper	6.80 × 10^−3^	1.72 × 10^−2^	1.68 × 10^−6^	3.85 × 10^−7^
Cucumber	8.00 × 10^−4^	7.93 × 10^−4^	9.07 × 10^−9^	2.09 × 10^−9^
Kale	1.20 × 10^−3^	4.29 × 10^−3^	7.35 × 10^−8^	1.69 × 10^−8^
Spinach	3.40 × 10^−3^	2.37 × 10^−3^	1.15 × 10^−7^	2.65 × 10^−8^
Cassava	9.00 × 10^−3^	7.71 × 10^−3^	9.92 × 10^−7^	2.28 × 10^−7^
Zucchini	6.80 × 10^−3^	5.71 × 10^−3^	5.54 × 10^−7^	1.28 × 10^−7^
Eggplant	6.80 × 10^−3^	1.33 × 10^−3^	1.29 × 10^−7^	2.97 × 10^−8^

* IR: Ingestion Rate; ** C: Concentration of Atrazine; *** EDI: Estimated Daily Intake, data available in Instituto Brasileiro de Geografia e Estatística (IBGE) 2020 [28].

**Table 3:** Main indicators of specific observed effects for each analyzed CAG2.

Food Item	Nervous System			Thyroid System HI
	Functional Effects on Motor Division HI	Functional Effects on Sensory Division HI	Functional Effects on Autonomic Division HI	
Orange	0.31	0.39	0.39	0.11
Strawberry	1.04	1.04	1.04	0.86
Grape	0.34	0.34	0.34	0.08
Papaya	0.21	0.21	0.21	0.00
Passionfruit	0.09	0.11	0.11	0.08
Apple	0.23	0.39	0.39	0.09
Peach	0.12	0.18	0.18	0.08
Pear	0.19	0.23	0.23	0.08
Mango	0.04	0.08	0.08	0.04
Guava	0.40	0.64	0.64	0.22
Tomato	0.41	0.46	0.46	0.38
Carrot	0.08	0.08	0.08	0.02
Sweet Potato	0.19	0.20	0.20	0.00
Sweet Pepper	0.55	0.56	0.56	0.38
Cucumber	0.04	0.07	0.07	0.38
Kale	0.28	0.29	0.29	0.27
Spinach	0.98	1.00	1.00	0.95
Cassava	2.99	3.15	3.16	2.77
Zucchini	0.37	0.37	0.54	0.19
Eggplant	0.40	0.40	0.40	0.21

## Data Availability

Data will be made available upon request.

## Supplementary Materials

The following supporting information can be downloaded at: https://www.mdpi.com/article/10.3390/foods15162868/s1, Table S1: Instrumental conditions for determining multiresidues of pesticides in food with high-water content; Table S2: Analytical figures of merit of the multi-residue method (QuEChERS-SPE/GC-MS). Values are expressed as mg kg^−1^; Table S3: Parameters used for the risk characterization; Table S4: Maximum residue levels for 11 pesticides in commonly consumed crops in Brazil (mg kg^−1^); Table S5: Main indicators of specific observed effects for each analyzed CAG2.
