# Supplementary material for: Cumulative Dietary Risk of Pesticide Mixtures in Plant-Based Foods: Beyond Single-Compound Regulatory Compliance
Source: Foods. 2026 Aug 17;15(16):2868. doi: 10.3390/foods15162868 (PMC13512862; doi:10.3390/foods15162868)
Supplement: Supplementary file 1 [file foods-15-02868-s001.zip › foods-4461766-supplementary.pdf]

Supplementary Information

Cumulative Dietary Risk of Pesticide Mixtures in Plant-Based Foods: Beyond Single-Compound Regulatory Compliance

Gabriel Henrique Savietto 1,*,† , Ana Beatriz Cintra Da Silva 1,†, Pedro Henrique Da Silveira Neves 1 , Lucas Silva Brito 1 , Bruno Alves Rocha 2 , Jonas Augusto Rizzato Paschoal 1 , Joaquim Rovira 3,4 , Jose L. Domingo 3 , Fernando Barbosa, Jr. 5,* and Marília Cristina Oliveira Souza 1

1 Department of Biomolecular Sciences, School of Pharmaceutical Sciences of Ribeirão Preto, University of São Paulo, Av. do Café s/nº, Ribeirão Preto 14040-903, SP, Brazil; mcosouza@usp.br (M.C.O.S.)

2 Institute of Chemistry, Federal University of Alfenas, Alfenas 37130-001, MG, Brazil

3 Laboratory of Toxicology and Environmental Health, School of Medicine, Universitat Rovira I Virgili, Sant Llorenç 21, 43201 Reus, CT, Spain

4 Institut de Recerca Biomèdica Catalunya Sud (IRB CatSud), 43204 Reus, CT, Spain

5 Department of Clinical Analyses, Toxicology, and Food Sciences, School of Pharmaceutical Sciences of Ribeirão Preto, University of Sao Paulo, Av. do Café s/nº, Ribeirão Preto 14040-903, SP, Brazil

* Correspondence: saviettog@usp.br (G.H.S.); fbarbosa@fcfrp.usp.br (F.B.J.)

† These authors contributed equally to this work.

Table S1. Instrumental conditions for determining multiresidues of pesticides in food with high-water content.

Classification	Pesticide	Retention time (min)	m/z
Organophosphate	Diazinon	8.26	137 – 179 – 304*
	Malathion	9.38	125 – 173*
	Pirimifos	8.31	168* - 304 – 318 – 333
	Chlorpyrifos	9.47	124 – 197* - 199 – 314
Carbamate	Carbofuran	7.69	122 – 149 – 164*
	Carbaryl	9.35	115 – 116 – 141 – 144*
	Pirimicarb	9.49	72 – 166* - 238
Herbicide	Atrazine	7.79	173 – 200* – 202 – 215
	2,4-D	8,.8	162* - 164 – 220
Neonicotinoid	Imidacloprid	9.09	99* – 126 – 211
Insecticides	Fipronil	9.48	213 – 367* – 399

*m/z used for the quantification processing method.

Table S2. Analytical figures of merit of the multi-residue method (QuEChERS-SPE/GC-MS). Values are expressed as mg kg^{-1}.

Compounds	Correlation coefficient (*r*) Linearity 0.001 – 10 mg/kg	Quality Control* (Spiked)	Intra-assay		Interassay		ME[c] (%)
			Precision[a]	Accuracy [b]	Precision	Accuracy	
Diazinon	0.9977	QCL – 0.05	4.88	97.19	10.45	102.91	8.79
		QCM – 4.00	0.80	112.33	13.77	100.44	
		QCH – 8.00	9.39	87.38	9.52	88.39	
Malathion	0.9982	0.05	3.92	107.28	6.01	107.44	9.55
		4.00	0.47	100.39	4.05	97.05	
		8.00	0.37	105.35	5.89	100.51	
Pirimifos	0.9932	0.05	0.24	114.27	6.22	111.10	11.18
		4.00	2.10	106.81	3.91	106.93	
		8.00	0.57	103.63	1.45	102.36	
Chlorpyrifos	0.9988	0.05	2.01	101.35	3.95	101.42	9.25
		4.00	1.26	104.32	4.02	102.25	
		8.00	1.19	98.98	0.91	99.48	
Carbofuran	0.9865	0.05	0.81	98.42	9.73	111.51	9.59
		4.00	8.22	100.62	11.43	102.72	
		8.00	4.19	102.63	7.34	99.83	
Carbaryl	0.9872	0.05	0.71	97.45	10.83	105.31	7.76
		4.00	9.22	110.72	10.93	112.84	
		8.00	3.49	100.33	4.23	102.63	
Pirimicarb	0.9988	0.05	0.93	93.56	12.77	105.17	10.39
		4.00	1.26	104.66	6.63	110.64	
		8.00	7.35	99.94	11.60	93.90	
Atrazine	0.9865	0.05	0.83	99.56	11.72	100.17	11.58
		4.00	1.36	102.65	7.65	109.64	
		8.00	7.44	98.94	12.61	96.90	
2,4-D	0.9834	0.05	1.34	111.67	9.67	111.55	8.79
		4.00	1.85	112.19	6.36	112.14	
		8.00	5.03	109.55	8.68	109.89	
Imidacloprid	0.9922	0.05	0.23	104.65	9.33	104.54	7.74
		4.00	0.84	109.18	3.31	111.16	
		8.00	4.02	108.52	6.69	104.88	
Fipronil	0.9871	0.05	1.44	105.69	9.44	106.56	10.94
		4.00	2.85	99.28	4.56	106.46	
		8.00	6.04	98.53	7.89	107.98	

[a] Precision: calculated by the coefficient of variation (CV %). [b] Accuracy: calculated by the percentage of imprecision (RSD %). [c] ME: Matrix effect
[*]Quality control samples: QCL: quality control of low concentration. QCM: quality control of medium concentration. QCH: quality control of high concentration.

Table S3. Parameters used for the risk characterization.

Compound	RfD[1]	SFe[2]
2,4-Dichlorophenoxyacetic acid	1.00E-02	-
Atrazine	3.00E-03	2.30E-01
Carbofuran	5.00E-03	-
Carbaryl	1.00E-01	-
Pirimicarb	-	-
Malathion	2.00E-02	-
Diazinon	7.00E-04	-
Pirimifos	7.30E-03	-
Chlorpyrifos	1.00E-03	-
Fipronil	2.00E-04	-
Imidacloprid	5.70E-02	-

[1]RfD: Chronic reference dose (mg/kg/day)
[2]SF: slope factor value $(mg/kg/day)^{-1}$

Table S4: Maximum residue levels for 11 pesticides in commonly consumed crops in Brazil (mg kg^{-1}).

LMR	2,4-D	Atrazine	Carbofuran	Carbaryl	Pirimicarb	Malathion	Diazinon	Pirimiphos	Chlorpyrifos	Fipronil	Imidacloprid
Orange	2	-	-	-	-	4	0.7	-	2	-	2
Strawberry	-	-	-	-	-	1	-	-	-	-	0.02
Grape	-	-	-	-	-	-	-	-	-	-	1
Papaya	-	-	-	-	-	-	-	-	-	-	2
Passionfruit	-	-	-	-	-	-	-	-	-	-	0.2
Apple	-	-	-	2	-	2	0.5	-	1	-	-
Peach	-	-	-	-	-	6	-	-	-	-	0.1
Pear	-	-	-	-	-	0.5	-	-	-	-	-
Mango	-	-	-	-	-	-	-	-	-	-	0.7
Guava	-	-	-	-	-	-	-	-	-	-	0.1
Tomato	-	-	-	0.1	1	3	-	-	0.5	-	0.5
Carrot	-	-	-	-	-	-	-	-	-	-	0.05
Sweet Potato	-	-	-	-	-	-	-	-	-	-	-
Sweet Pepper	-	-	-	-	-	-	-	-	-	-	0.5
Cucumber	-	-	-	0.02	1	3	-	-	-	-	0.2
Kale	-	-	-	-	1	3	-	-	-	-	2
Spinach	-	-	-	-	-	-	-	-	-	-	-
Cassava	-	-	-	-	-	-	-	-	-	-	-
Zucchini	-	-	-	-	-	-	-	-	-	-	0.05
Eggplant	-	-	-	-	1	0.5	-	-	-	-	0.5

Table S5: Main indicators of specific observed effects for each analyzed CAG2.

Compound	Nervous system			Thyroid system
	Functional effects on motor division	Functional effects on sensory division	Functional effects on autonomic division	
2,4-D	-	-	Urination	Increased relative thyroid weight
Atrazine	-	-	-	-
Carbofuran	Reduced grip strength, landing foot splay	*n/s	n/s	-
Carbaryl	Tremor, erythrocyte and brain AChE inhibition	Decreased pupil size	Salivation	Follicular cell hypertrophy/hyperplasia
Pirimicarb	Tremor	n/s	Urination, salivation	-
Malathion	n/s	n/s	n/s	-
Diazinon	Reduced forelimb and handlimp grip strength	n/s	n/s	-
**Pirimiphos	-	n/s	n/s	-
Chlorpyrifos	Decreased motor activity	n/s	n/s	-
Fipronil	Convulsions	Hiperreactivity	n/s	Increased relative thyroid weight
Imidacloprid	Tremor	n/s	n/s	-

Where there is a "-" there was no specific indicator of effect but the mechanism of action was still noted in the EFSA document, including NOAEL and/or LOAEL values.
*n/s: Not sufficient data regarding the health impact.
**Based on pirimiphos-ethyl data according to the Read-Across approach.
